# SMADs binding site polymorphisms rs9911630 is associated with susceptibility but not prognosis of gastric cancer: a case control study

**DOI:** 10.7150/jca.40089

**Published:** 2020-06-01

**Authors:** Liyang Liu, Ming Lu, Xi Gu, Xiang Ma, Jiaxi Feng, Yang Cao, Weida Gong, Qinghong Zhao, Fulin Qiang

**Affiliations:** 1 Department of General Surgery, the Second Affiliated Hospital of Nanjing Medical University, Nanjing, Jiangsu, China.; 2 Department of General Surgery, Yixing Tumor Hospital, Yixing, Jiangsu, China.; 3 Department of Core Laboratory, Nantong Tumor Hospital, Nantong, Jiangsu, China.

**Keywords:** rs9911630, polymorphisms, susceptibility, gastric cancer

## Abstract

**Background:** Single nucleotide polymorphisms (SNPs) in transcription factor binding sites (TFBS) can change their binding strength, affecting the function of transcription factors (TFs). Small mother against decapentaplegic (SMAD) proteins are known as a family of TFs involved in tumorigenesis. We performed this study to investigate whether SNPs in SMADs binding sites affect the susceptibility or prognosis of gastric cancer (GC).

**Methods:** Using bioinformatics tools, we focused on the association between rs9911630 polymorphism and GC. We performed this case-control study in 1275 GC patients and 1426 cancer-free subjects using TaqMan allelic discrimination method.

**Results:** We found that rs9911630 A>G polymorphism was associate to an increased risk of gastric cancer (adjusted OR for additive model = 1.16; 95% CI = 1.03-1.30). Furthermore, we assess whether rs9911630 polymorphism affected the prognosis of GC. However, no significant association was discovered between rs9911630 A>G polymorphism and overall survival time of GC patients (HR for addictive model = 1.01; 95%CI = 0.88-1.15).

**Conclusions:** Our results suggested that rs9911630 polymorphism in SMADs target site might influence susceptibility but not prognosis of gastric cancer.

## Introduction

Gastric cancer (GC) is the fourth most common cancer and second dominant cause of cancer-related death worldwide 1,2. In spite of major improvements in diagnosis and treatment, the 5-year survival rate of GC is still less than 25% 3. Therefore, it is urgently required to identify a new way for predicting GC susceptibility and prognosis 4-6. Both environmental and genetic factors are involved in etiology of GC. Environmental risk factors such as older age, *Helicobacter pylori* infection and tobacco smoking nowadays are well-known for their role in GC 7. Pathogenetic mechanisms in GC are still being debated but in recent years, a number of single nucleotide polymorphisms (SNPs) have been found playing a vital role in gastric carcinogenesis 8.

Transcription factor (TF) dysregulation, playing a vital role in abnormal gene expression, is a hallmark of many cancers 9,10. The genomic locations of TF binding at specific locus have functional consequences with respect to the binding ability of TF 11. SNPs seating in transcriptional factors binding sites (TFBS) may conclusively influence the binding ability and modulate individual cancer susceptibility 12-14. In addition, studies indicated that SNPs may modify the methylation level of gene promoter regions, interfering with TF binding, which in turn leads to abnormalities of gene transcription 15,16. The identification of these SNPs that represent a functional link with methylation sites provided functional insight into the potential mechanism by which genetic variants involved in etiology of tumor.

Small mother against decapentaplegic (SMAD) proteins, as a family of transcription factors, are expressed broadly in the body tissues 17. SMAD proteins act as mediators of transforming growth factor-beta (TGF-β) signaling, which is one of the most important tumor suppressor pathways 18. SMADs translocate signals from the cell surface to the nucleus, regulating TGF-β superfamily-dependent gene expression 19. The TGF-β/SMAD signaling pathway was found to regulate cell growth and promotes apoptosis of epithelial cells, and participate in angiogenesis 20. Accumulating evidence indicated that components of this pathway are involved in a large range of cancers 21-23. Function of this signaling pathway may be influenced when a genetic variant occurs in the SMADs' binding site. We evaluated the associations between these SNPs and GC susceptibility using GWAS data. Among all these eligible SNPs, we found rs9911630 A>G could affect the methylation level of CpG sites in promoter regions of three genes. So, we selected rs9911630 and evaluated its effect on the susceptibility and prognosis of GC in this study.

## Methods

### Study population

There were 1,275 GC cases and 1,426 age- and sex-matched cancer-free controls covered in our study. All cases were supported by the Cancer Clinical Research Base of Nanjing Medical University between March 2006 and May 2013. Only histologically confirmed GC patients were included. Exclusion criteria included secondary GC or metastasized cancer from other organs. In addition, patients that received neoadjuvant chemotherapy or radiotherapy before surgery were excluded. All control subjects were randomly enrolled at the same period when they sought physical examinations in the same hospital. The controls were frequency-match to cases on age (±5 years) and sex. All patients enrolled in this study were genetically unrelated ethnic Han Chinese. The study was authorized by the institutional review board of Nanjing Medical University. Every participant enrolled in this study signed an informed consent.

### SNPs selection

SNPs located in SMADs binding sites were searched according to genotype data of genome-wide association studies (GWASs). Then we evaluated the associations between these SNPs and GC susceptibility using GWAS data and identified eligible SNPs with a standard of *P* < 0.01 and minor allele frequency (MAF) > 0.05. A total of 556 relevant SNPs were obtained from GWAS datasets, and after the process of our selection, 8 eligible SNPs were taken into further consideration (**Table 1**). We would like to focus on SNPs acting as methylation Quantitative Trait Loci (meQTLs) in the surrounding region. The level 3 Human Methylation 450 and Level 2 SNP Array data of gastric adenocarcinoma were downloaded from the The Cancer Genome Atlas (TCGA) database. We tested the methylation status of CpG sites situated within 10000 bases range of each SNP by meQTL analysis. Finally, rs9911630 were enrolled in further study.

### SNPs genotyping

We isolated genomic DNA from peripheral blood. The selected SNPs were genotyped using TaqMan allelic discrimination assay on the ABI 7900HT Real-Time PCR System (Applied Biosystems, Foster City, CA, USA). For confirmation, 10% of the samples were selected to be genotyped again, and the results were in consistent with the first assay. The structure of primers and probes are as follows: forward primer: 5'- GCTCTCTAAGGTCCCTTCTCATTG-3', reverse primer: 5' -GCACAAGTGACCGATGGGTAA-3', and probes: FAM: AAGCACAGTGCATGGA, HEX-AAGCACAGCGCATG.

### Statistical analysis

We assessed the differences in demographic factors by Student's t test and Pearson's chi-squared (χ^2^) test. Hardy-Weinberg equilibrium (HWE) of the controls was assessed by a goodness-of-fit χ^2^ test. The ORs and 95% CIs were calculated to estimate associations between these SNPs and GC susceptibility. Variables of age and sex were used as covariates adjusted for the association analysis. We used multiple inheritance models to estimate the significance of SNP rs9911630. Kaplan-Meier method and log-rank test were applied to evaluate the associations between survival time and the SNP rs9911630. Mean survival time was provided when the median survival time (MST) could not be calculated. We performed Univariate or multivariate Cox regression analysis to calculated crude or adjusted hazard ratios (HRs) and 95% CIs. *P* < 0.05 for two-side Student's t test was considered statistically significant when analyzing the promoter activity. All statistical analyses were carried out using SAS software (version 9.1.3; SAS Institute Inc., Cary, NC, USA).

## Results

### Association between SNP rs9911630 and GC risk from publicly databases

We downloaded publicly available GWAS datasets from dbGaP database. We used additive model to evaluate the association between SNP rs9911630 and GC risk. As a result, we found rs9911630 A>G polymorphism were significantly associated with GC risk (adjusted OR = 1.11, 95% CI = 1.01-1.23, *P* < 0.01). We performed meQTL analysis based on TCGA datasets to test whether these SNPs are associated with the methylation status of CpG sites situated nearby. As shown in **Table 1**, rs9911630 A>G was related to methylation level of CpG sites in promoter regions of three genes (the neighbor of brca1 gene, the breast and ovarian cancer susceptibility gene 1 and long intergenic non- coding RNA 910). rs9911630 G allele was related to the decreased methylation level of cg10047753 and cg23758822 (*P* = 2.68×10^-30^ for cg10047753 and *P* = 2.43×10^-26^ for cg23758822). Besides, rs9911630 G allele was related to the increased methylation level of other six CpG sites (*P* = 1.70×10^-16^ for cg05368731, *P* = 1.68×10^-12^ for cg19454999, *P* = 8.45×10^-7^ for cg25072359, *P* = 1.19×10^-5^ for cg25918947, *P* = 8.50×10^-5^ for cg01879757, *P* = 1.42×10^-5^ for cg25067162).

### Characteristics of cases and controls

Then we perform a case-control study to further evaluate the associations between SNP rs9911630 and GC susceptibility using our samples. In this study, no remarkable difference was found among cases and controls in the distributions of age (*P* = 0.324) and sex (*P* = 0.358). Clinicopathological characteristics of case-control studies were summarized in **Table 2**. Of these cases, there were 61.3% non-cardia gastric cancer patients, and 33.6% cardia gastric cancer patients; 682 (61.4%) had lymph node metastasis and 167 (15.1%) existed distant metastasis. In addition, all the cases were identified to the TNM stage in accordance with the 6^th^ edition staging manual of the American Joint Committee on Cancer (AJCC). TNM stage I, II, III, and IV were with the percentage of 23.1%, 24.6%, 35.5%, and 16.8%, respectively.

### Association of rs9911630 polymorphism with gastric cancer risk

Genotype distributions rs9911630 among the patients and controls were shown in **Table 3**. The genotype frequencies were agreed with the Hardy-Weinberg equilibrium (*P* = 0.1166). Different inheritance models were used and the results indicated that rs9911630 A>G polymorphism were significantly associated with GC risk in additive models (adjusted OR = 1.16, 95% CI = 1.03-1.30, *P* = 0.012); codominant model (adjusted OR for GG genotype = 1.39, 95% CI = 1.09-1.78, *P* = 0.009) and recessive model (adjusted OR = 1.32, 95% CI = 1.05-1.66, *P* = 0.020). As result, rs9911630 G allele was a potential risk allele for GC. The main findings of this case-control study were consistent with analysis based on publicly databases.

### Stratified analysis of SNP rs9911630 and GC risk

We analyzed the effects of rs9911630 polymorphism on GC risk stratified in accordance with different clinical variables. As shown in **Table 4**, we found association between rs9911630 G allele and increased risk of GC among subgroup of non-cardia (adjusted OR = 1.21, 95%CI = 1.06-1.38,* P* = 0.004) and histological types of diffuse (adjusted OR = 1.27, 95%CI = 1.11-1.47, *P* = 0.001). Significant risk effect was not observed in subgroups of different depth of invasion, lymph node metastasis, distant metastasis or TNM stages.

### SNP rs9911630 polymorphism and gastric cancer survival

Since rs9911630 G allele was a potential risk allele for GC, we would like to assess the prognostic value of SNP rs9911630 on GC patients. This study comprised 933 patients with gastric cancer and overall survival was the end point. Characteristics and clinical features of subjects were summarized in **Table 5**. Histology, the depth of invasion, lymph node status, distant metastasis and TNM stage were factors affecting the survival time of GC patients (log-rank *P <* 0.05).

We used log-rank test to evaluate the effect of rs9911630 A>G on overall survival time in GC patients. However, as the presented in **Table 6,** significant association was not observed between the rs9911630 polymorphism and overall survival time in additive model (log-rank *P* = 0.691), dominant model (log-rank *P* = 0.630) or recessive model (log-rank *P* = 0.612). To further assess the association between the rs9911630 and survival of patients with GC, we performed subgroup analyses by clinical characteristics under dominant model. There was no prominent association between SNP rs9911630 polymorphism and survival time when stratified by age, sex, tumor site, histology, depth of invasion, lymph node metastasis, distant metastasis, TNM stage and chemotherapy (**Table 7**). As a result, we did not find significant association between rs9911630 and GC prognosis in the present study.

## Discussion

As a family of transcription factors, SMADs may impact regulation of target genes, participating in cancer-related biological processes 24,25. The abnormal expression of SMADs was found in several human malignancies, including GC 26,27. Wu et al. 28 have reported that genetic variations in SMAD4 gene are related to GC susceptibility. Recently, many evidences have been documented between TFBS and GC pathogenesis. Hence, SNPs in SMADs binding sites are expected to become risk markers for GC.

In this study, we studied one SNP (rs9911630 A>G) lying in the binding site of SMADs to explore its association with GC susceptibility and survival. Firstly, we found SNP rs9911630 was related to GC risk according to publicly databases. Then we perform a study of 1,275 cases and 1,426 controls to further evaluate the associations between SNP rs9911630 and GC susceptibility. Results showed that rs9911630 can influence the risk of GC, and individuals with the rs9911630 variant genotypes (GG) had observably increased GC risk compared with those with the AA/AG genotypes. However, significantly association was not observed between rs9911630 A>G polymorphism and overall survival time of GC patients. In addition, we found rs9911630 G allele was associated with increased risk of non-cardia GC but not cardia GC. Many SNPs have been reported to be susceptibility locus specific for cardia GC or for non-cardia GC 29. Gastric cardia carcinoma differs from non-cardia carcinoma in epidemiological characteristics, etiology and clinical features. Risk factors for gastric cardia adenocarcinomas also differ between these two main sub-locations of GC. Studies in western populations have put forward that cardia adenocarcinomas are more similar to esophageal adenocarcinomas 30. SNPs in a locus on chromosome 10q23 in the PLCE1 gene were reported to have strong association with gastric cardia adenocarcinoma and esophageal squamous cell carcinoma, but no association with gastric non-cardia adenocarcinoma 31. These findings suggested that identification of phenotype-specific genetic susceptibility loci could improve understanding of different subtypes of GC, which in turn is important for early detection, diagnosis and treatment of this malignancy.

In the present study, we did not perform functional study to estimate the role of rs9911630 polymorphism in the current study. However, we performed meQTL analysis based on TCGA datasets, and as a result, we found that rs9911630 A>G was associated with methylation level of CpG sites in promoter regions of three genes (the neighbor of brca1 gene, the breast and ovarian cancer susceptibility gene 1 and long intergenic non- coding RNA 910). DNA methylation plays an important role in modulating the transcription of mammalian genomes by blocking the binding of transcription factors. Previous studies have demonstrated that SNPs may modify the methylation level of CpG sites or influence the generation of new CpG sites, which changes the status of genes' methylation and regulate gene expression 32-34. Accordingly, we speculated that the SNP rs9911630 could influence the binding ability of SMADs and change methylation level of the gene promoter regions nearby, which in turn leads to the influence of gene outputs. Despite the exact mechanism remained to be elucidated, these functions of SNP rs9911630 may play roles in gastric carcinogenesis.

Trying the best of ourselves with existing materials, this is the first study exploring the association between the SNP rs9911630 polymorphism of SMAD*s* binding site and susceptibility and prognosis of GC. However, the current study was subject to limitations. Firstly, some environmental factors like smoking, drinking and *Helicobacter pylori* infection play vital roles in gastric carcinogenesis, but due to the unavailability of detailed information in some of the study subjects, we did not perform a further analysis to investigate the gene-environment interaction. Secondly, sample size of the current study is small, making analysis less reliable than if a large sample had been available. Thirdly, functional study was not operated to estimate the role of rs9911630 polymorphism.

## Conclusions

In conclusion, our results suggested that rs9911630 polymorphism in SMADs target site might influence susceptibility but not prognosis of GC in the Chinese populations. Meanwhile, methylation level of the nearby gene promoter regions could be changed according to the polymorphism rs9911630, which might influence the expression of these genes. Larger, well-designed epidemiologic and functional studies are still needed to prove these findings.

## Figures and Tables

**Table 1:** Characteristics of the selected SNPs

SNP	Gene	Allele	MAF	OR (95% CI)^a^	meQTL (risk allele association, P)
rs17707882	MYO10	C>T	0.148	0.84 (0.73-0.96)	cg18061395, *P* =1.31×10^-4^(decreased) cg24556395, *P* = 4.22×10^-5^(decreased)
rs9353563	CNR1	A>G	0.246	1.11 (1.01-1.24)	cg02436141, *P* =2.63×10^-4^(decreased) cg18703951, *P* =4.34×10^-3^(decreased) cg08458400, *P* =1.85×10^-2^(decreased) cg23276695, *P* =3.57×10^-2^(increased)
rs1569836	AGPAT4	A>G	0.275	1.14 (1.03-1.26)	cg02031769, *P* =2.25×10^-3^(decreased) cg11697870, *P* =1.73×10^-2^(decreased)
rs10514486	SLC36A4	T>C	0.397	0.90 (0.82-0.99)	cg15554438, *P* =2.96×10^-2^(decreased)
rs9911630	LINC0091(lncRNA), NBR1, BRCA1	A>G	0.334	1.11 (1.01-1.23)	cg10047753, *P* =2.68×10^-30^(LINC00910, decreased) cg23758822, *P* =2.43×10^-26^(LINC00910, decreased) cg05368731, *P* =1.70×10^-16^(NBR1, increased) cg19454999, *P* =1.68×10^-12^(NBR1, increased) cg25072359,* P* =8.45×10^-7^(NBR1, increased) cg25918947, *P* =1.19×10^-5^(NBR1, increased) cg01879757, *P* =8.50×10^-5^(BRCA1, increased) cg25067162, *P* =1.42×10^-5^(BRCA1, increased)
rs618443	CCNY	G>A	0.375	1.16 (1.06-1.28)	cg05845615,* P* =2.65×10^-2^(decreased)
rs4940826	LMAN1	A>G	0.21	1.12 (1.00-1.26)	cg25361621,* P* =1.55×10^-4^(increased)
rs10423232	ANKLE1	T>C	0.324	0.89 (0.81-0.99)	cg00433770, *P* =2.39×10^-3^(increased)

^a^Adjusted by age and sex in logistic additive analysis.

**Table 2:** Characteristics of study subjects

Variables	Cases		Controls		*P*^a^
	N=1275	%	N=1426	%	
Age (years) (mean±SD)	63.1±10.7		63.3±11.0		0.595
**Sex**					0.347
Male	880	69.0	963	67.5	
Female	392	30.8	463	32.5	
NA	3	0.2			
**Tumor site**					
Cardia	403	33.6			
Non-cardia	734	61.3			
Both	61	5.1			
NA	77				
**Histological types**					
Intestinal	513	45.6			
Diffuse	612	54.4			
NA	150				
**Depth of invasion**					
Tis	1	0.1			
T1	170	15.2			
T2	169	15.1			
T3	575	51.5			
T4	204	18.1			
NA	158				
**Lymph node metastasis**					
N0	428	35.6			
N1/N2/N3	682	61.4			
NA	165				
**Distant metastasis**					
M0	941	84.9			
M1	167	15.1			
NA	167				
**TNM**					
I	267	23.1			
II	284	24.6			
III	410	35.5			
IV	194	16.8			
NA	120				

Two-sided student t test for the frequency distributions of age between the cases and controls. Two-sided χ^2^ test for the frequency distributions of sex between the cases and controls.

**Table 3:** Association of rs9911630 polymorphism with gastric cancer risk

	Genotype	Cases/controls	OR (95% CI)	Adjusted OR (95% CI)^a^	*P^a^*
Additive model	AA	497/603	1.15 (1.03-1.29)	**1.16 (1.03-1.30)**	**0.012**
	AG	603/669			
	GG	175/154			
Codominant model	AA	497/603	1.00	1.00	
	AG	603/669	1.09 (0.93-1.29)	1.11 (0.94-1.30)	0.227
	GG	175/154	1.38 (1.08-1.77)	**1.39 (1.09-1.78)**	**0.009**
Dominant model	AA	497/603	1.00	1.00	
	AG/GG	778/823	1.15 (0.98-1.34)	1.16 (0.99-1.35)	0.062
Recessive model	AA/AG	1100/1272	1.00	1.00	
	GG	175/154	1.32 (1.04-1.66)	**1.32 (1.05-1.66)**	**0.020**

^a^Adjusted by age and sex in logistic additive analysis.

**Table 4:** Associations between rs9911630 genotypes and clinical characteristics of GC

Variables	OR (95% CI)	Adjusted OR (95% CI)^a^	*P^a^*
Controls	1.00	1.00	
**Tumor site**			
Cardia	1.01 (0.85-1.19)	1.02 (0.86-1.20)	0.835
Non-cardia	**1.21 (1.06-1.38)**	**1.21 (1.06-1.38)**	**0.004**
**Histological types**			
Diffuse	**1.26 (1.10-1.45)**	**1.27 (1.11-1.47)**	**0.001**
Intestinal	1.02 (0.88-1.18)	1.02 (0.87-1.19)	0.825
**Depth of invasion**			
T1	1.25 (0.99-1.58)	1.25 (0.99-1.59)	0.063
T2	1.10 (0.87-1.40)	1.12 (0.88-1.42)	0.367
T3	1.08 (0.94-1.25)	1.08 (0.94-1.25)	0.283
T4	1.12 (0.90-1.40)	1.13 (0.90-1.41)	0.291
**Lymph node metastasis**			
N0	1.10 (0.94-1.30)	1.11 (0.95-1.31)	0.196
N1/N2/N3	1.12 (0.98-1.29)	1.12 (0.98-1.29)	0.097
**Distant metastasis**			
M0	1.10 (0.97-1.24)	1.10 (0.97-1.24)	0.128
M1	1.22 (0.97-1.53)	1.25 (0.99-1.58)	0.056
**TNM stages**			
I+II	1.12 (0.97-1.30)	1.12 (0.97-1.30)	0.123
III+IV	1.12 (0.97-1.29)	1.13 (0.98-1.30)	0.098

^a^Adjusted by age and sex in logistic additive analysis.

**Table 5:** Patients' characteristics and clinical features

Variables	Patients (n=933)	Deaths (n=439)	MST (months)	Log-rank *p*	Adjusted HR (95% CI)
**Age**					
≤60	436	201	90.1	0.285	1.00
>60	497	235	60.0		1.11 (0.92-1.34)
**Sex**					
Male	718	332	75.5	0.412	1.00
Female	215	104	64.3		1.10 (0.88-1.37)
**Site**					
Cardia	356	165	66.9	0.580	1.00
Non-cardia	577	271	71.0		1.06 (0.87-1.28)
**Histology**					
Diffuse	536	280	51.3	0.001	1.00
Intestinal	397	156	57.6^a^		**0.72 (0.59-0.88)**
**Depth of invasion**					
*T*1	149	45	48.7^a^	<0.001	1.00
*T*2	199	83	90.1		**1.54 (1.07-2.21)**
*T*3	540	284	49.2		**2.15 (1.57-2.95)**
*T*4	45	27	26.9		**2.77 (1.72-4.46)**
**Lymph node metastasis**					
*N*0	372	130	83.1^a^	<0.001	1.00
*N*1-*N*3	561	306	44.4		**1.87 (1.52-2.29)**
**Distant metastasis**					
*M*0	875	401	75.5	0.003	1.00
*M*1	58	35	27.5		**1.67 (1.18-2.36)**
**TNM stage**					
I+II	259	87	60.8^a^	<0.001	1.00
III+IV	578	294	54.5		**1.79 (1.41-2.27)**
**Chemotherapy**					
No	629	299	75.1	0.728	1.00
Yes	304	137	61.5		1.04 (0.85-1.27)

^a^Mean survival time was provided when MST could not be calculated.

**Table 6:** Association between rs9911630 and overall survival of GC

SNP	Genetic models	Genotypes	All cases	Deaths	MST	Log-rank *p*	HR (95% CI)^a^
rs9911630	addictive	AA	350	163	82.1	0.691	1.01 (0.88-1.15)
		AG	439	210	60.0		
		GG	144	63	66.9		
	dominant	AA	350	163	82.1	0.630	1.00
		AG/GG	583	273	63.5		1.06 (0.87-1.29)
	recessive	AA/AG	789	373	71.0	0.612	1.00
		GG	144	63	66.9		0.93 (0.71-1.21)

^a^Adjusted for age and sex.

**Table 7:** Subgroup analyses of association between rs9911630 polymorphisms and GC survival

Variables	Genotype (deaths/patients)		HR (95% CI)^a^
	AA	AG/GG	
Total	163/350	273/583	1.06(0.87-1.29)
**Age**			
≤60	154/235	124/201	0.91(0.69-1.21)
>60	156/262	149/235	1.19(0.92-1.56)
**Sex**			
Male	239/386	203/332	1.02(0.82-1.28)
Female	71/111	70/104	1.183(0.785-1.783)
**Site**			
Cardia	121/191	998/165	0.953(0.698-1.301)
Non-Cardia	189/306	175/271	1.124(0.876-1.443)
**Histology**			
Diffuse	159/256	166/280	0.957(0.753-1.217)
Intestinal	151/241	107/156	1.333(0.950-1.871)
**Depth of Invasion**			
T1	67/104	29/45	0.974(0.524-1.809)
T2	68/117	56/82	1.422(0.891-2.267)
T3	164/258	172/282	0.975(0.767-1.240)
T4	11/18	16/27	1.140(0.508-2.558)
**Lymph node metastasis**			
N0	143/242	81/130	1.157(0.811-1.651)
N1-N3	167/255	192/306	0.986(0.781-1.246)
**Distant metastasis**			
M0	293/474	248/401	1.053(0.860-1.289)
M1	17/23	25/35	1.258(0.571-2.771)
**TNM stage**			
I-II	104/172	56/87	1.187(0.762-1.850)
III-IV	177/284	181/294	1.032(0.815-1.307)
**Chemotherapy**			
No	201/330	181/299	1.045(0.828-1.318)
Yes	109/167	92/137	1.104(0.771-1.579)

^a^Adjusted for age and sex.

## Abbreviations

SNPs: single nucleotide polymorphisms (SNPs)
TFBS: transcription factor binding sites
TFs: transcription factors
SMAD: small mother against decapentaplegic
GC: gastric cancer
TGF-β: transforming growth factor-beta
GWASs: data of genome-wide association studies
MAF: minor allele frequency
meQTLs: methylation Quantitative Trait Loci
TCGA: The Cancer Genome Atlas
HWE: Hardy-Weinberg equilibrium
MST: median survival time
